# Type I interferon receptor controls B-cell expression of nucleic acid-sensing Toll-like receptors and autoantibody production in a murine model of lupus

**DOI:** 10.1186/ar2771

**Published:** 2009-07-22

**Authors:** Donna L Thibault, Kareem L Graham, Lowen Y Lee, Imelda Balboni, Paul J Hertzog, Paul J Utz

**Affiliations:** 1 Department of Medicine, Division of Immunology and Rheumatology, Stanford University School of Medicine, 269 Campus Drive, CCSR 2250, Stanford, CA, 94305, USA; 2 Current address: Genentech, Inc., 1 DNA Way, South San Francisco, CA 94080, USA; 3 Department of Pediatrics, Division of Pediatric Rheumatology, Stanford University School of Medicine, 300 Pasteur Drive, Boswell Building A085, Stanford, CA, 94305, USA; 4 Centre for Functional Genomics and Human Disease, Monash Institute of Medical Research, 27-31 Wright Street, Clayton, Victoria 3168, Australia

## Abstract

**Introduction:**

Systemic lupus erythematosus (SLE) is a chronic autoimmune disease characterized by the production of high-titer IgG autoantibodies directed against nuclear autoantigens. Type I interferon (IFN-I) has been shown to play a pathogenic role in this disease. In the current study, we characterized the role of the IFNAR2 chain of the type I IFN (IFN-I) receptor in the targeting of nucleic acid-associated autoantigens and in B-cell expression of the nucleic acid-sensing Toll-like receptors (TLRs), TLR7 and TLR9, in the pristane model of lupus.

**Methods:**

Wild-type (WT) and IFNAR2^-/- ^mice were treated with pristane and monitored for proteinuria on a monthly basis. Autoantibody production was determined by autoantigen microarrays and confirmed using enzyme-linked immunosorbent assay (ELISA) and immunoprecipitation. Serum immunoglobulin isotype levels, as well as B-cell cytokine production *in vitro*, were quantified by ELISA. B-cell proliferation was measured by thymidine incorporation assay.

**Results:**

Autoantigen microarray profiling revealed that pristane-treated IFNAR2^-/- ^mice lacked autoantibodies directed against components of the RNA-associated autoantigen complexes Smith antigen/ribonucleoprotein (Sm/RNP) and ribosomal phosphoprotein P0 (RiboP). The level of IgG anti-single-stranded DNA and anti-histone autoantibodies in pristane-treated IFNAR2^-/- ^mice was decreased compared to pristane-treated WT mice. TLR7 expression and activation by a TLR7 agonist were dramatically reduced in B cells from IFNAR2^-/- ^mice. IFNAR2^-/- ^B cells failed to upregulate TLR7 as well as TLR9 expression in response to IFN-I, and effector responses to TLR7 and TLR9 agonists were significantly decreased as compared to B cells from WT mice following treatment with IFN-α.

**Conclusions:**

Our studies provide a critical link between the IFN-I pathway and the regulation of TLR-specific B-cell responses in a murine model of SLE.

## Introduction

Autoantibodies directed against nucleic acid-associated autoantigens are characteristic of the autoimmune disease systemic lupus erythematosus (SLE). The role of the type I interferon (IFN-I) system in the pathogenesis of both human and murine SLE has been studied extensively (reviewed in [1]). Many SLE autoantigens contain nucleic acids and act as endogenous ligands for nucleic acid-sensing Toll-like receptors (TLRs) [2]. Ligation of TLR9 by DNA-associated autoantigens or TLR7 by RNA-associated autoantigens induces secretion of IFN-I by plasmacytoid dendritic cells (PDCs) and activates autoreactive B cells [3-12]. Production of anti-DNA autoantibodies requires TLR9, and the production of anti-ribonucleoprotein (anti-RNP) autoantibodies requires TLR7 [13,14]. A duplication of the TLR7 gene in *Yaa *mice is sufficient for the induction of autoantibodies against RNA-associated targets [15,16], although some studies suggest that other genes in this locus contribute to autoimmunity in this model [17,18]. TLRs control isotype switching to pathogenic IgG isotypes in SLE as MyD88^-/- ^and TLR9^-/- ^SLE mice lack autoantibodies of the IgG2a and IgG2b subclasses [19].

Mice treated with a single intraperitoneal injection of the mineral oil pristane develop a lupus-like disease characterized by the production of autoantibodies directed against many lupus autoantigens, including DNA/histones and components of the U1 small nuclear RNP (snRNP)/Smith antigen (Sm) complex [20]. Autoantibodies directed against this complex are associated with both human and murine lupus [21], and the RNA component can serve as an endogenous ligand for TLR7 [3,5,6,8-10]. Importantly, pristane-treated TLR7^-/- ^mice fail to develop isotype-switched anti-snRNP/Sm autoantibodies [14]. Pristane treatment results in the formation of lipogranulomas and the overexpression of IFN-inducible genes [22], which closely resembles the IFN-I-induced gene expression signature seen in blood cells derived from human patients with SLE [23,24] and is dependent on TLR7 [25]. In addition, treatment with pristane induces apoptosis *in vivo*, providing a potential source of autoantigens [26], including RNPs and nucleosomes.

All subtypes of IFN-I bind to the IFN-I receptor (IFNAR), which is composed of two chains: IFNAR1 and IFNAR2. The IFNAR2 chain exists in both transmembrane and soluble isoforms and is critical for ligand binding and signal transduction through the receptor [27,28]. Negative regulators of IFN and other proinflammatory cytokine signaling, including suppressor of cytokine signaling 1 (SOCS1) and the Tyro-3, Axl, and Mer (TAM) receptors, have been shown to associate with, and regulate signaling through, the IFNAR1 chain [29,30]. Signaling through the IFNAR results in activation of the IFN-stimulated gene factor 3 (ISGF3) heterotrimeric complex, composed of STAT1, STAT2, and IFN regulatory factor 9 (IRF9) [31]. We have previously shown that the IFN-I signaling molecules IRF9 and STAT1 are required for the production of IgG autoantibodies in the pristane model and mediate the IFN-I-inducible expression of TLR7 and TLR9 in B cells [32]. We also noted a requirement for these molecules for isotype switching to the pathogenic IgG2a isotype in this model. Nacionales and colleagues [33] demonstrated that mice deficient in the IFNAR1 chain of the receptor fail to develop anti-Sm/RNP and anti-chromatin autoantibodies in the pristane model, although TLR responses were not characterized in these mice. Also, isotype analysis of antigen-specific autoantibodies was not performed. Interestingly, pristane-treated IFNAR1^-/- ^mice produced normal serum levels of IgG2a, and a high percentage developed anti-nuclear autoantibodies (ANAs).

In the present study, we characterized the role of the IFNAR2 chain of the IFNAR in the pristane model. Pristane-treated IFNAR2^-/- ^mice developed high titers of total serum IgM accompanied by significantly lower levels of the pathogenic IgG2a isotype. Pristane-treated IFNAR2^-/- ^mice failed to develop IgG autoantibodies directed against both RNA- and DNA-associated autoantigens. TLR7 expression and activation by TLR7 agonists were completely abolished in IFNAR2^-/- ^B cells, demonstrating that B-cell activation through TLR7 requires IFNAR2. In addition, B cells from IFNAR2^-/- ^mice failed to upregulate TLR9 expression and activation following incubation with IFN-I. Our results demonstrate a novel role for the IFNAR2 chain of the IFNAR in TLR7- and TLR9-specific B-cell responses and in the production of autoantibodies directed against nucleic acid-associated targets.

## Materials and methods

### Mice and treatment

BALB/cJ mice were purchased from The Jackson Laboratory (Bar Harbor, ME, USA). IFNAR2^-/- ^mice on the BALB/c background were provided by Paul J Hertzog (Monash University, Clayton, Australia) [30]. Mice were maintained under standard conditions at the Stanford University Research Animal Facility. Female mice 8 to 10 weeks of age were given a single 0.5 mL intraperitoneal injection of pristane (Sigma-Aldrich, St. Louis, MO, USA) or phosphate-buffered saline (PBS). Sera were collected before injection and at 4-week intervals. Proteinuria was monitored by dipstick analysis using Albustix (Bayer Corp., Elkhart, IN, USA) on a monthly basis. All animal experiments were approved by, and performed in compliance with, the guidelines of the Institutional Animal Care and Use Committee.

### Autoantigen microarrays

Antigens were printed in ordered arrays on FAST slides (Whatman, now part of GE Healthcare, Piscataway, NJ, USA). Arrays were blocked with PBS containing 3% fetal bovine serum (FBS) and 0.05% Tween-20 (Sigma-Aldrich) overnight at 4°C. Arrays were probed with 1:300 dilutions of mouse serum for 1 hour at 4°C followed by washing and incubation with a 1:2,000 dilution of cyanine 3-conjugated goat anti-mouse (GAM)-IgG/IgM (Jackson ImmunoResearch Laboratories, Inc., West Grove, PA, USA). Arrays were scanned using a GenePix 4000B scanner (Molecular Devices Corporation, Sunnyvale, CA, USA). The median pixel intensities of individual features were determined using GenePix Pro version 6.0, and background values were subtracted. The data were expressed as normalized median net digital fluorescence units, representing median values from eight replicate features on each array normalized to the median intensity of eight GAM-Ig features. Significance analysis of microarrays (SAM) [34] was applied to the dataset. A hierarchical clustering algorithm [35] using the uncentered correlation similarity metric and complete linkage method was applied, and results were depicted as a heatmap and dendogram generated using Java Treeview software [36]. A full list of antigens included on the array and detailed protocols are provided [see Additional data file 1] [37].

### Enzyme-linked immunosorbent assays

For anti-single-stranded DNA (anti-ssDNA) enzyme-linked immunosorbent assays (ELISAs), Nunc MaxiSorp plates (Nalgene, a brand of Thermo Scientific Nunc, Rochester, NY, USA) were coated with 10 μg/mL calf thymus DNA (Sigma-Aldrich). For anti-Sm/RNP and anti-ribosomal phosphoprotein P0 (anti-RiboP) ELISAs, plates were coated with 1 μg/mL Sm/RNP or RiboP (Diarect AG, Freiburg, Germany). Wells were incubated with sera diluted 1:250 in PBS containing 3% FBS and 0.05% Tween-20 followed by incubation with horseradish peroxidase (HRP)-conjugated GAM-IgM or GAM-IgG (SouthernBiotech, Birmingham, AL, USA). Tetramethylbenzidine (Pierce, Rockford, IL, USA) was added, and optical density values were determined at 450 nm.

To determine levels of total serum Ig isotypes, plates were coated with 5 μg/mL GAM-Ig (H+L) (SouthernBiotech) overnight at 4°C. Wells were incubated with 1:5,000,000 dilution for IgG, or 1:500,000 dilution for all other isotypes, of sera in PBS containing 3% FBS and 0.05% Tween-20 followed by isotype-specific HRP-conjugated GAM-Ig (SouthernBiotech). Standard curves were constructed using mouse Ig isotype standards (SouthernBiotech), and total levels were determined.

### Real-time quantitative polymerase chain reaction

Splenocytes were harvested from age- and gender-matched wild-type (WT) and IFNAR2^-/- ^mice. B cells were negatively selected using magnetic beads (Miltenyi Biotec, Bergisch Gladbach, Germany). Cells were more than 95% pure, as assessed by flow cytometry (B220^+^biotin^-^; data not shown). B cells were cultured in RPMI supplemented with L-glutamine (2 mM), sodium pyruvate (1 mM), nonessential amino acids (0.1 mM), penicillin (100 U/mL), streptomycin (0.1 mg/mL), 2-ME (5 × 10^-5 ^M), and FBS (10%) in the presence or absence of 1,000 IU/mL recombinant IFN-α (Calbiochem, now part of EMD Biosciences, Inc., San Diego, CA, USA) for 4 hours. RNA was extracted using RNeasy Mini kit (Qiagen Inc., Valencia, CA, USA). RNA (10 ng) was amplified using one-step QuantiTect SYBR Green reverse transcription-polymerase chain reaction (Qiagen Inc.) and 0.5 μM forward and reverse primers using an Opticon2 continuous fluorescence detector (MJ Research, now part of Bio-Rad Laboratories, Inc., Hercules, CA, USA). The fold change in expression of each transcript normalized to glyceraldehyde-3-phosphate dehydrogenase (GAPDH) was determined using the 2^-ΔΔCt ^method. QuantiTect Primer Assay sets for murine TLR7, TLR9, and GAPDH were purchased from Qiagen Inc.

### Proliferation assay

Splenocytes were harvested at the conclusion of the study 12 months following pristane injection, and B cells were purified as above. Cells were stimulated with 1 μM ODN1826 or 1 mM Loxoribine (InvivoGen, San Diego, CA, USA). Sixteen hours following stimulation, wells were pulsed with 1 μCi [^3^H]TdR (Amersham, now part of GE Healthcare) and harvested 24 hours following stimulation. Incorporated radioactivity was measured using a betaplate scintillation counter.

### Interleukin-6 production

B cells were purified, cultured, and stimulated as above. After 24 hours in culture, supernatants were assayed for production of interleukin-6 (IL-6) by sandwich ELISA using a commercially available ELISA kit (BD Pharmingen, San Diego, CA, USA). For IFN-α pretreatment studies, B cells were incubated in the presence or absence of 1,000 IU/mL IFN-α for 24 hours. TLR ligands were then added as above, and IL-6 concentration in the supernatant was determined 24 hours following stimulation.

## Results

### Proteinuria

To address the role of IFN-I in the development of autoimmunity in the pristane model of SLE, WT and IFNAR2^-/- ^mice were treated with either pristane or PBS as a negative control. WT BALB/c mice treated with pristane develop an immune complex-mediated glomerulonephritis [38]. The development of proteinuria in the mice, a measure of kidney disease, was therefore assessed. Over the course of 12 months, 5 of 10 (50%) pristane-treated WT mice developed proteinuria, whereas none of 10 (0%) pristane-treated IFNAR2^-/- ^mice developed proteinuria (Table 1). These data suggest that IFN-I signaling through IFNAR2 is critical for the development of kidney damage in the pristane model of SLE. Because the development of kidney disease in the pristane model is not as severe as in other spontaneous models of SLE, such as the (NZB × NZW)F1 or the MRL/*lpr *models, we focused our studies instead on the mechanisms of autoantigen selection and on the role of IFN-I and TLRs in this process.

**Table 1:** Development of proteinuria

Genotype	Treatment	Number	Proteinuria^a ^(percentage)
WT	PBS	5	0 (0)
WT	Pristane	10	5 (50)
IFNAR2^-/-^	PBS	4	0 (0)
IFNAR2^-/-^	Pristane	10	0 (0)^b^

^a^Proteinuria is at least 300 mg/dL. ^b^*P *< 0.05 versus wild-type (WT) pristane, Fisher exact test. IFNAR2, interferon-I receptor 2; PBS, phosphate-buffered saline.

### Hypergammaglobulinemia

**Figure 1:**
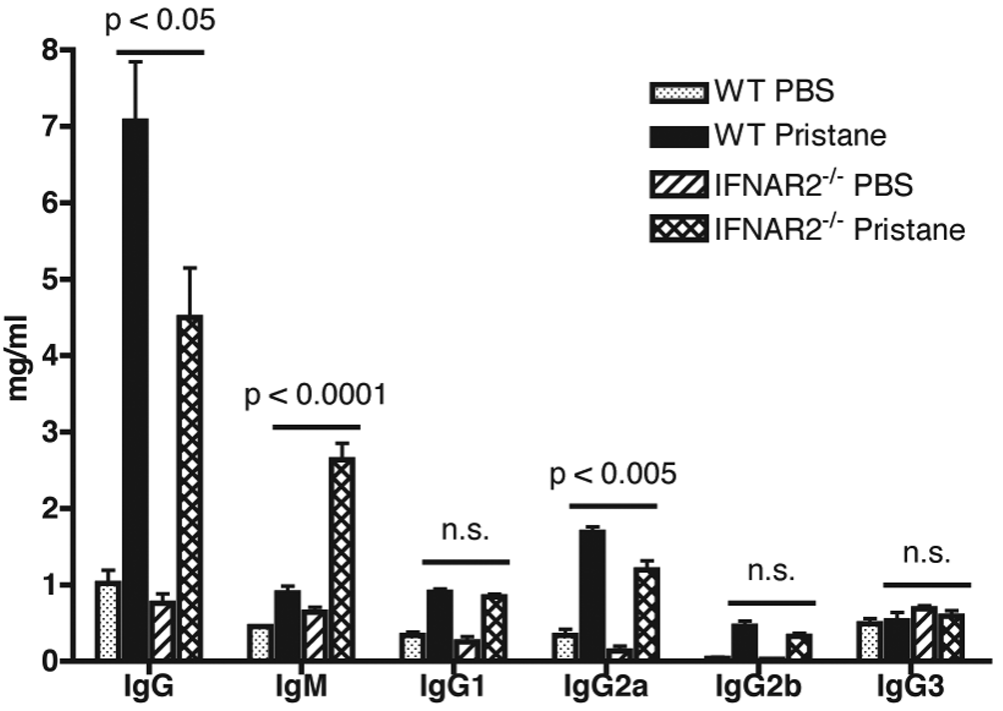
Serum immunoglobulin levels in pristane-treated mice. Total immunoglobulin levels were measured by enzyme-linked immunosorbent assay in serum obtained 6 months after treatment with phosphate-buffered saline (PBS) or pristane. Mean values with standard deviation are shown for each group. *P *values were obtained using the Student *t *test and are displayed above each plot. IFNAR2: interferon-I receptor 2; n.s.: not significant; WT: wild-type.

Following pristane treatment, WT mice develop hypergammaglobulinemia characterized by the production of high levels of IgG as well as increased levels of IgM [39]. Importantly, pristane induces the production of high levels of IgG2a, a pathogenic isotype that preferentially binds the activating Fc receptor, FcγRIV [40]. IFN-I induces B-cell maturation and promotes isotype switching to all subclasses of IgG [41,42]. We examined the production of immunoglobulin isotypes in pristane-treated IFNAR2^-/- ^mice (Figure 1). Consistent with the known role of IFN-I in isotype switching, pristane-treated IFNAR2^-/- ^mice had significantly higher levels of total serum IgM and significantly lower levels of total serum IgG when compared with pristane-treated WT mice. In contrast to the phenotype seen in IFNAR1^-/- ^mice [33], pristane-treated IFNAR2^-/- ^mice developed significantly lower levels of the pathogenic isotype IgG2a as compared with pristane-treated WT mice. There were no significant differences in the levels of IgG1, IgG2b, or IgG3 between pristane-treated WT and IFNAR2^-/- ^mice.

### Autoantibody production

**Figure 2:**
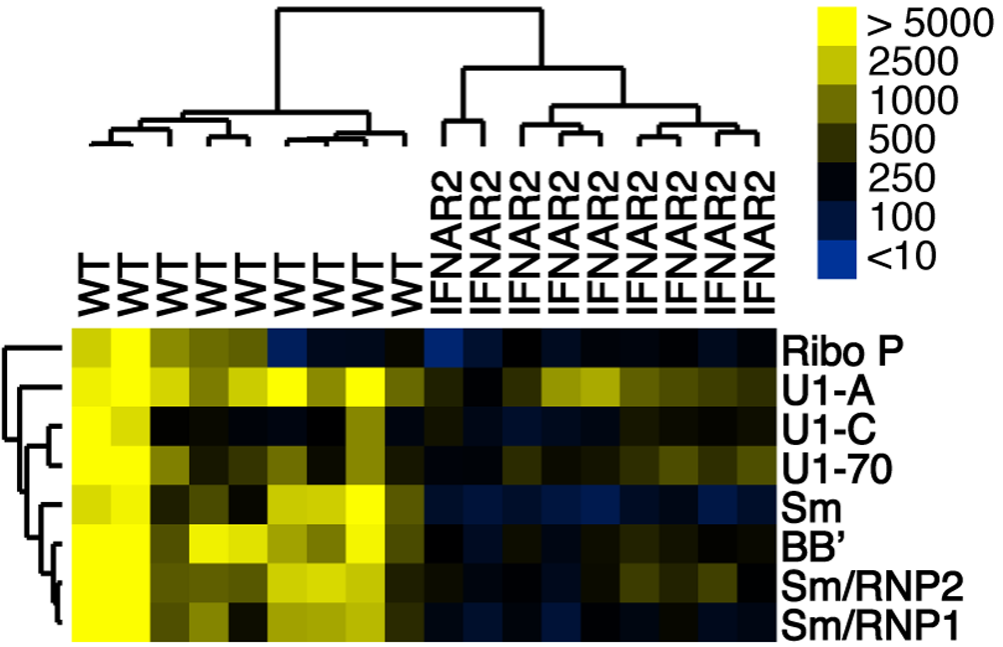
Autoantibody profiling of pristane-treated mice using autoantigen microarrays. Individual arrays composed of over 50 recombinant or purified antigens were incubated with diluted sera obtained 6 months after pristane treatment. Pairwise significance analysis of microarrays was used to determine antigen features with statistically significant differences in array reactivity between pristane-treated wild-type (WT) and pristane-treated IFNAR2^-/- ^mice (false discovery rate < 0.05, fold change > 3). IFNAR2: interferon-I receptor 2; RiboP: ribosomal phosphoprotein P0; Sm: Smith antigen; SmRNP: Smith antigen ribonucleoprotein.

We have used autoantigen microarrays to profile the autoantibody response in murine models of SLE [32,43-45] and in humans with rheumatic diseases [46,47]. We employed this technique to systematically profile the autoantibody response in pristane-treated WT and IFNAR2^-/- ^mice. Serum from individual mice was used to probe lupus autoantigen microarrays that contained more than 50 candidate SLE autoantigens. A table containing raw median pixel intensity minus background values for all array antigens is provided [see Additional data file 2]. We used the SAM algorithm [34] to determine statistically significant differences in array reactivity between pristane-treated WT and IFNAR2^-/- ^mice followed by hierarchical clustering [35] to order individual mice on the basis of similarity of autoantibody profiles directed against the significant antigens identified by SAM. The results are displayed as a heatmap (Figure 2). SAM identified reactivity to components of two RNA-containing complexes as significantly different between these two groups. Autoantibodies that recognize components of the U1-snRNP complex (Sm/RNP, Sm, BB', U1-A, U1-C, U1–70) and ribosomal P (RiboP) were present in pristane-treated WT mice but were significantly decreased in pristane-treated IFNAR2^-/- ^mice. The two groups of mice separated into completely distinct clusters based on autoantibody reactivity to these autoantigens.

We frequently employ autoantigen microarrays as a screening tool to identify autoantibody reactivities using a multiplex platform and rely heavily on statistical algorithms to determine significant differences. Reactivities to all autoantigens are then validated using conventional techniques such as immunoprecipitation, ELISA, and Western blot. WT mice treated with pristane develop high-titer autoantibodies capable of immunoprecipitating the Sm/RNP complex from radiolabeled cell extract [20]. As anticipated, serum autoantibodies from 7 of 10 (70%) WT mice treated with pristane immunoprecipitated components of this complex; however, none of 10 (0%) pristane-treated IFNAR2^-/- ^mice developed these antibodies (Table 2). These results confirm the specific lack of autoantibodies directed against the Sm/RNP complex in serum from pristane-treated IFNAR2^-/- ^mice, confirming the data obtained using autoantigen microarrays.

**Table 2:** Immunoprecipitation of the Smith antigen/ribonucleoprotein complex

Genotype	Treatment	Number	Sm/RNP (percentage)
WT	PBS	5	0 (0)
WT	Pristane	10	7 (70)
IFNAR2^-/-^	PBS	4	0 (0)
IFNAR2^-/-^	Pristane	10	0 (0)^a^

^a^*P *< 0.005 versus wild-type (WT) pristane, Fisher exact test. IFNAR2, interferon-I receptor 2; PBS, phosphate-buffered saline; Sm/RNP, Smith antigen/ribonucleoprotein.

**Figure 3:**
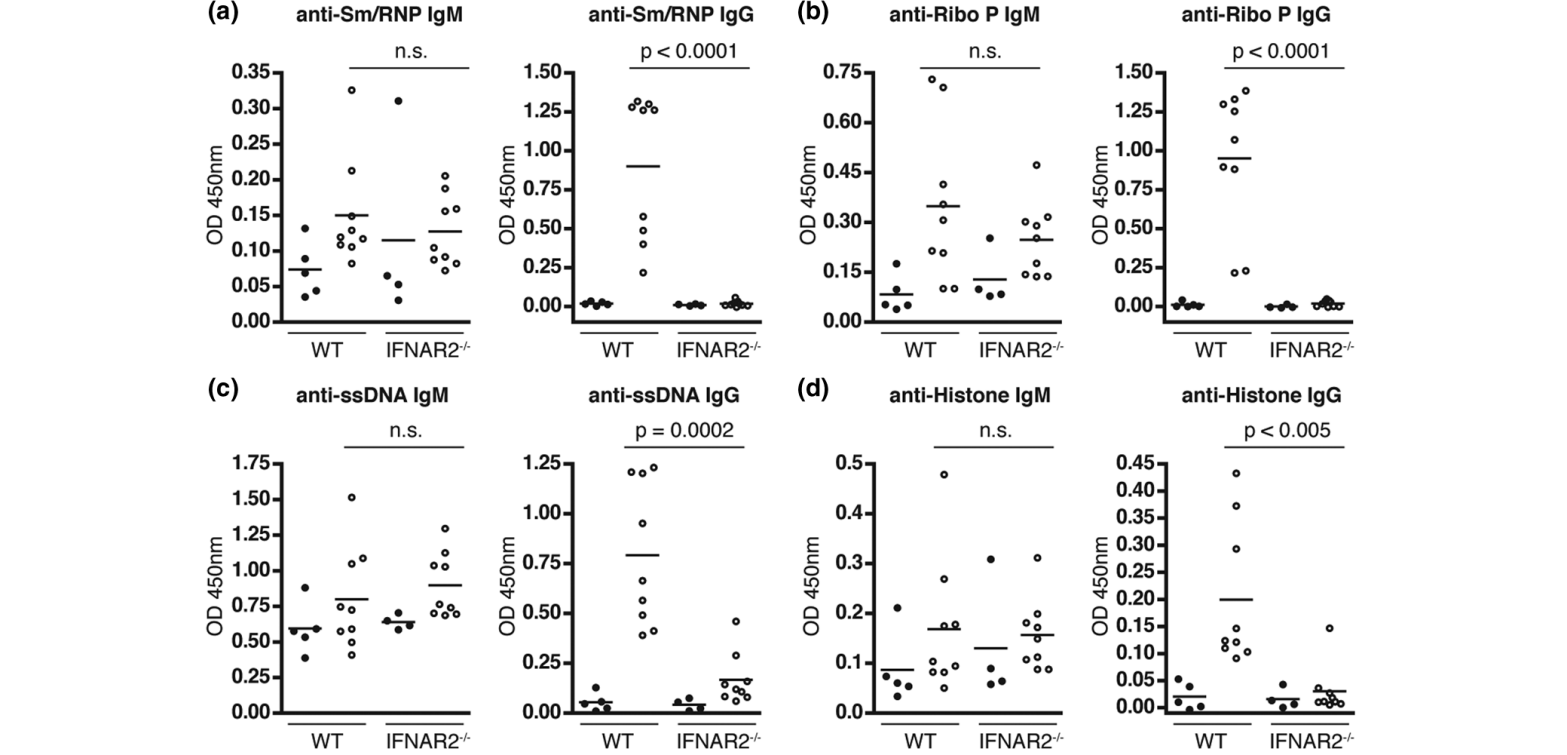
Autoantibody production in pristane-treated IFNAR2^-/- ^mice. Sera obtained 6 months after treatment with pristane or phosphate-buffered saline (PBS) were analyzed for levels of IgM or IgG anti-Sm/RNP **(a)**, anti-RiboP **(b)**, anti-ssDNA **(c)**, or anti-Histone **(d) **antibodies by enzyme-linked immunosorbent assay. Data are plotted as absorbance values for individual animals minus background. *P *values were determined using the Mann-Whitney *t *test for pristane-treated wild-type (WT) versus pristane-treated IFNAR2^-/- ^mice and are displayed above each graph. Closed circles represent serum from PBS-treated mice, and open circles represent serum from pristane-treated mice. IFNAR2: interferon-I receptor 2; n.s.: not significant; OD: optical density; RiboP: ribosomal phosphoprotein P0; Sm/RNP: Smith antigen/ribonucleoprotein; ssDNA: single-stranded DNA.

Our previous studies have demonstrated that the IFN-I downstream signaling molecule, IRF9, was required for the production of IgG autoantibodies directed against the RNA-associated targets, Sm/RNP and RiboP, as well as against the DNA-associated targets, ssDNA and histones. Despite failing to produce IgG autoantibodies, pristane-treated IRF9^-/- ^mice developed significantly higher titers of IgM autoantibodies directed against the two RNA-associated complexes [32]. We therefore examined the production of IgG and IgM autoantibodies directed against these targets in IFNAR2^-/- ^mice (Figure 3). Consistent with the microarray data, IFNAR2 is absolutely required for the development of IgG anti-Sm/RNP (Figure 3a, right panel) and anti-RiboP (Figure 3b, right panel) autoantibodies. In contrast to the phenotype seen for IRF9^-/- ^mice, however, pristane-treated IFNAR2^-/- ^mice do not develop significantly higher titers of IgM autoantibodies directed against either of these targets as compared with pristane-treated WT mice (Figures 3a and 3b, left panels). WT mice treated with pristane develop high titers of IgG anti-ssDNA (Figure 3c, right panel) and anti-histone (Figure 3d, right panel) autoantibodies. Pristane-treated IFNAR2^-/- ^mice develop significantly lower titers of IgG autoantibodies directed against these two targets (Figures 3c and 3d). There are no significant differences in levels of IgM anti-ssDNA (Figure 3c, left panel) or anti-histone (Figure 3d, left panel) between pristane-treated WT and IFNAR2^-/- ^mice. These data demonstrate that IFNAR2 is absolutely required for the development of IgG autoantibodies directed against all of the major antigenic targets in the pristane model of SLE: Sm/RNP, RiboP, and the nucleosome.

### Toll-like receptor expression

**Figure 4:**
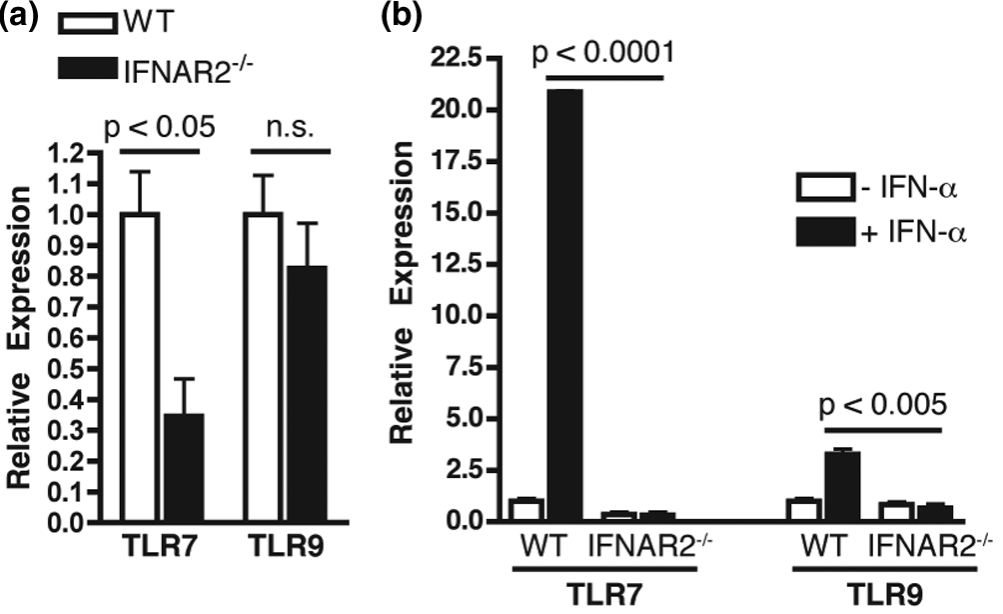
Expression of Toll-like receptors TLR7 and TLR9 in IFNAR2^-/- ^B cells. **(a) **B cells were purified from wild-type (WT) or IFNAR2^-/- ^mice using magnetic beads. RNA was extracted and the relative mRNA expression of TLR7 and TLR9 was measured. **(b) **Purified B cells were cultured in the presence or absence of interferon-alpha (IFN-α). RNA was extracted and the relative expression TLR7 and TLR9 was measured. *P *values were determined using the Student *t *test. IFNAR2: interferon-I receptor 2; n.s.: not significant.

PDC secretion of IFN-α has been shown to enhance the expression of TLR7 in human naïve B cells [48]. In support of this study, we have previously reported a critical role for the IFN-I signaling components IRF9 and STAT1 in murine B-cell expression of TLR7 as well as in the IFN-I-mediated induction of TLR9 expression [32]. We examined the mRNA expression levels of these TLRs in B cells from IFNAR2^-/- ^mice. IFNAR2^-/- ^B cells expressed lower basal levels of TLR7 when compared with WT B cells; however, there was no significant difference in the expression of TLR9 (Figure 4a). As demonstrated previously, the expression of TLR7 in B cells from WT mice was induced more than 20-fold following treatment with IFN-α (Figure 4b). This induction of TLR7 expression was completely dependent on IFNAR2 as there was no change in TLR7 expression in B cells from IFNAR2^-/- ^mice following treatment with IFN-α. The expression of TLR9 in WT B cells was upregulated approximately 3-fold upon treatment with IFN-α and this upregulation was also completely dependent on IFNAR2 (Figure 4b). IFNAR2 is therefore required for the induction of TLR7 and TLR9 expression in B cells in response to IFN-α and for normal basal levels of B-cell TLR7 expression.

### Toll-like receptor activation

**Figure 5:**
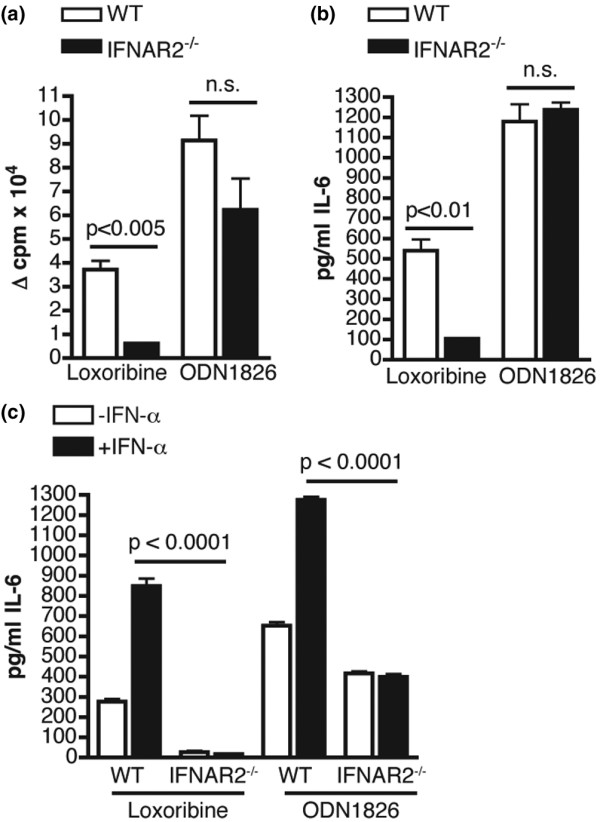
**Activation of Toll-like receptors TLR7 and TLR9 in IFNAR2^-/- ^mice**. **(a) **B cells were purified from pristane-treated wild-type (WT) or IFNAR2^-/- ^mice, and proliferation in response to Loxoribine or ODN1826 was measured. Data are represented as the difference in mean counts per minute (cpm) of stimulated and unstimulated triplicate wells (Δ cpm) + standard error of the mean. **(b) **B cells were purified as above and the concentration of interleukin-6 (IL-6) in the supernatant was measured following stimulation with Loxoribine or ODN1826. **(c) **B cells were purified as above and were cultured in the presence or absence of interferon-alpha (IFN-α) for 24 hours before treatment with Loxoribine or ODN1826. The concentration of IL-6 in the supernatant was then measured. *P *values were determined using the Student *t *test. IFNAR2: interferon-I receptor 2; n.s.: not significant; ODN: oligodeoxynucleotide.

We next examined the functional ability of B cells from pristane-treated IFNAR2^-/- ^mice to respond to TLR7 and TLR9 agonists. B cells from pristane-treated WT and IFNAR2^-/- ^mice were cultured with the TLR7 agonist, Loxoribine, or the CpG motif-containing TLR9 agonist, ODN1826. IFNAR2^-/- ^B cells proliferated significantly less (Figure 5a) and secreted significantly less IL-6 (Figure 5b) versus WT B cells in response to Loxoribine. Consistent with basal expression data, there were no significant differences in proliferation (Figure 5a) or IL-6 secretion (Figure 5b) in response to the TLR9 agonist in B cells from pristane-treated IFNAR2^-/- ^mice.

Because IFN-α upregulated B-cell expression of TLR7 and TLR9, we examined the ability of IFN-α to enhance B-cell activation by TLR ligands. B cells from WT mice pretreated with IFN-α secreted significantly more IL-6 than untreated WT B cells (*P *= 0.0001) in response to Loxoribine (Figure 5c). In striking contrast, B cells from IFNAR2^-/- ^mice secreted very low levels of IL-6 in response to Loxoribine, and this was not enhanced by pretreatment with IFN-α (*P *< 0.0001 versus IFN-α-treated WT B cells, Figure 5c). Although IFNAR2^-/- ^B cells responded normally to the TLR9 agonist in the absence of exogenous IFN-α (Figure 5b), the IFN-α-mediated enhancement of B-cell activation by ODN1826 was completely abolished in B cells from IFNAR2^-/- ^mice (Figure 5c). These studies indicate that IFN-I signaling through IFNAR2 mediates both the expression of, and activation through, nucleic acid-sensing TLRs in B cells.

## Discussion

Previously, we have demonstrated that the IFN-I signaling molecules, IRF9 and STAT1, were required for the production of IgG autoantibodies in the pristane model and for the high expression levels of TLR7 and TLR9 following treatment with IFN-I in B cells [32]. Here, we describe the autoantibody profile and TLR-dependent B-cell response in SLE mice genetically deficient in the IFNAR2 chain of the IFNAR. Autoantibody profiling using autoantigen microarrays in combination with conventional techniques to confirm the array results revealed that, similar to the phenotype for IRF9^-/- ^mice, pristane-treated IFNAR2^-/- ^mice specifically lacked IgG autoantibodies directed against all of the major targets in the pristane model. These targets included components of the RNA-associated complexes Sm/RNP and RiboP as well as the DNA-associated autoantigens ssDNA and histones. B cells from IFNAR2^-/- ^mice exhibited defects in the expression of TLR7 as well as in responses to TLR7 agonists in the absence of exogenous IFN-α. Upon treatment with IFN-α, B cells from WT mice upregulated TLR7 expression over 20-fold, upregulated TLR9 expression approximately 3-fold, and secreted significantly higher levels of IL-6 in response to stimulation through either TLR7 or TLR9. In the absence of IFNAR2, however, this IFN-α-mediated enhancement of TLR7 and TLR9 expression and activation was completely abolished. TLR7 responses, in particular, were almost undetectable. Taken together with our studies in IRF9^-/- ^mice, the results of these experiments demonstrate a critical role for the IFN-I pathway in the activation of B cells and subsequent autoantibody production in response to TLR agonists. We are currently in the process of backcrossing the IRF9^-/-^, STAT1^-/-^, and IFNAR2^-/- ^genetic deletions onto the MRL/*lpr *background in order to more carefully assess the role of this molecule in the development of lupus nephritis and to determine whether other major autoantigen classes are still targets of autoantibodies.

There are three very important differences between the phenotypes observed for IRF9^-/- ^and IFNAR2^-/- ^mice in the pristane model. First, pristane-treated IRF9^-/- ^mice developed significantly higher titers of IgM autoantibodies directed against the RNA-associated autoantigens Sm/RNP and RiboP [32]. This phenotype was not observed in IFNAR2^-/- ^mice as there was no significant difference in levels of IgM autoantibodies directed against these two targets versus WT mice treated with pristane or versus PBS-treated IFNAR2^-/- ^mice (Figures 3a and 3b). Second, although the expression of TLR7 was significantly decreased in IRF9^-/- ^B cells versus WT B cells following treatment with IFN-α, TLR7 expression in IFN-α-treated IRF9^-/- ^B cells was actually significantly increased versus untreated IRF9^-/- ^B cells [32]. This was not the case for IFNAR2^-/- ^B cells as TLR7 expression was not induced, even at lower levels, following treatment with IFN-α (not significant versus untreated IFNAR2^-/- ^B cells, Figure 5b). The small increase in expression in IRF9^-/- ^B cells has functional implications as IFN-α-treated IRF9^-/- ^B cells secreted significantly more IL-6 versus untreated IRF9^-/- ^B cells in response to a TLR7 agonist [32], whereas virtually no IL-6 was secreted by IFNAR2^-/- ^B cells in response to a TLR7 agonist, regardless of whether they were treated with IFN-α (Figure 5). Our studies therefore suggest that the IRF9-independent induction of TLR7 by IFN-I may be sufficient to drive the partial activation of B cells, which results in the production of high levels of IgM autoantibodies directed against RNA-associated targets. It is not sufficient, however, to drive the full activation of these cells to differentiate into isotype-switched IgG-secreting plasma cells. On the other hand, by inhibiting the IFN-I response further upstream through the IFNAR2 chain of the receptor, we observed a complete block in B-cell expression of TLR7, activation through TLR7, and autoantibody production directed against RNA-associated targets. Third, IRF9^-/- ^mice treated with pristane developed fatal plasmacytomas as early as 6 months following pristane injection, whereas no IFNAR2^-/- ^mice developed this phenotype. Because the majority of the IRF9^-/- ^mice developed this fatal condition prior to the conclusion of the study, we were unable to accurately assess kidney damage in this strain. As none of the IFNAR2^-/- ^mice developed any signs of proteinuria over the course of the 12-month study, we can now conclude that IFN-I signaling is crucial for the development of end-organ pathogenesis in this model.

The block in isotype switching to IgG in IRF9^-/- ^mice was restricted to TLR-dependent antigens as IRF9^-/- ^mice immunized with ovalbumin (OVA) in complete Freund's adjuvant, a strong stimulus, mounted an effective IgG anti-OVA response [32]. Although higher levels of IgM-specific autoantibodies were not observed in the pristane-treated IFNAR2^-/- ^mice, total serum levels of IgM were highly elevated in pristane-treated IFNAR2^-/- ^mice (Figure 1), suggesting that there may be defects in isotype switching to IgG in these mice. Total serum IgM levels were also increased in pristane-treated IFNAR1^-/- ^mice [33]. Studies in IFNAR1^-/- ^mice have revealed that IFN-I promotes isotype switching to all subtypes of IgG [41,42], although in the pristane model, total serum levels of IgG2a were normal in IFNAR1^-/- ^mice [33]. Future studies in IFNAR2^-/- ^mice are therefore aimed at investigating the role that IFNAR2 plays in isotype switching in B cells *in vitro *and in response to different TLR agonists *in vivo*.

Two key negative regulators of TLR responses have been found to physically associate with the IFNAR1 chain of the receptor: SOCS1 and the TAM receptors, which include Tyro, Axl, and Mer. The induction of the SOCS proteins by IFN-α is dependent on the TAM receptors [29] and both SOCS1^-/- ^and TAM receptor triple-knockout mice develop spontaneous lupus-like autoimmunity [49,50]. The expressions of the TAM receptors themselves are upregulated by IFN and TLR signaling. Both of these pathways require the presence of IFNAR1 and STAT1 [29]. Therefore, in addition to mediating signals initiated by IFN-α, IFNAR1 is critical for TAM receptor-mediated negative regulation of pleiotropic TLR responses. The function of TAM receptors has not been assessed in IFNAR2^-/- ^mice, although signals transduced by IFNAR2 are not influenced by SOCS1 *in vivo *[30]. We hypothesize that the lack of negative regulatory molecule function in IFNAR1^-/- ^mice may result in phenotypic differences between IFNAR1^-/- ^and IFNAR2^-/- ^mice in models of autoimmunity. Such differences are notable in the pristane model as IFNAR1^-/- ^mice developed high serum titers of the pathogenic IgG2a isotype and high ANA titers, although the identity of the autoantigen driving this response is unknown [33]. It will therefore be critical to assess the function of TAM receptors and the differential roles of IFNAR1 and IFNAR2 in the development of autoimmunity.

Unlike other murine models of SLE, such as the MRL/*lpr *and the (NZB × NZW)F1 spontaneous models, the pristane model is ideally suited for studying the IFN-I pathway in the development of murine SLE. This is true for several reasons. First, pristane injection has been shown to induce the accumulation of an IFN-producing Ly6C-high monocyte population [51], which drives the subsequent expression of IFN-I-inducible genes [22]. These same genes are overexpressed in blood cells from human lupus patients, and expression of these genes correlates with the production of anti-nucleoprotein autoantibodies [23,24,52-54]. In contrast, the expression of IFN-γ-regulated, but not IFN-I-regulated, genes is enhanced in both splenocyte subsets and kidneys of MRL/*lpr *mice, suggesting that the type II IFN pathway rather than the IFN-I pathway plays the dominant pathogenic role in the development of autoimmunity in this model. Second, the spectrum of autoantibodies produced upon pristane treatment represents several clinically assayed specificities in human SLE patients [55]. The (NZB × NZW)F1 model is inadequate to study the anti-RNP response as these animals do not develop autoantibodies directed against RNA-associated autoantigens, although pristane treatment of (NZB × NZW)F1 mice induces the production of these autoantibodies [56]. Finally, pristane induces apoptosis both *in vitro *and *in vivo*, providing a potential source of autoantigens [26]. Defects in clearance of apoptotic debris is a common feature of human SLE [57]. Therefore, disease pathogenesis in the pristane model recapitulates several key features of human SLE, including kidney pathology, IFN-I pathway activation, autoantibody production, and induction of apoptosis.

## Conclusions

In summary, our data demonstrate a novel role for the IFNAR2 in TLR7- and TLR9-specific B-cell responses and in the generation of IgG autoantibody responses *in vivo*. We propose that the production of IFN-I by DCs upon pristane treatment [22] induces the expression of TLR7 and TLR9 in B cells, resulting in the activation of autoreactive B cells and in autoantibody production *in vivo*. This response is completely dependent on signaling through IFNAR2. Our results provide further support for the development of specific inhibitors of TLR7, TLR9, and IFN-I signaling for the treatment of SLE in human patients and suggest that patients may be selected for such therapeutic approaches and monitored for response to therapy based on the targeting of subsets of nucleic acid-associated autoantigens. These studies are of particular importance given that IFN-I and TLR inhibitors are already being tested in SLE in early-phase human clinical trials. Our studies provide a crucial link between the IFN-I system and TLR signaling *in vivo *and suggest that IFN-I is upstream of TLRs in the loss of B-cell tolerance to nucleic acid-associated autoantigens in SLE.

## Abbreviations

ANA: anti-nuclear autoantibody; ELISA: enzyme-linked immunosorbent assay; FBS: fetal bovine serum; GAM-Ig: goat-anti-mouse-immunoglobulin; GAPDH: glyceraldehyde-3-phosphate dehydrogenase; HRP: horseradish peroxidase; IFN-I: type I interferon; IFNAR: interferon-I receptor; IL-6: interleukin-6; IRF9: interferon regulatory factor 9; ODN: oligodeoxynucleotide; OVA: ovalbumin; PBS: phosphate-buffered saline; PDC: plasmacytoid dendritic cell; RiboP: ribosomal phosphoprotein P0; RNP: ribonucleoprotein; SAM: significance analysis of microarrays; SLE: systemic lupus erythematosus; Sm: Smith antigen; snRNP: small nuclear ribonucleoprotein; SOCS1: suppressor of cytokine signaling 1; ssDNA: single-stranded DNA; TAM: Tyro-3, Axl, and Mer; TLR: Toll-like receptor; WT: wild-type.

## Competing interests

In the past 5 years, PJU has served as a consultant to Centocor, Inc. (Horsham, PA, USA), Biogen Idec (Cambridge, MA, USA), Avanir Pharmaceuticals (Aliso Viejo, CA, USA), Amgen (Thousand Oaks, CA, USA), UCB (Brussels, Belgium), Argos Therapeutics, Inc. (Durham, NC, USA), AstraZeneca (London, UK), CoMentis, Inc. (South San Francisco, CA, USA), Gilead Sciences, Inc. (Foster City, CA, USA), REGiMMUNE Corporation (Mountain View, CA, USA), Johnson & Johnson (New Brunswick, NJ, USA), and Genentech, Inc. (South San Francisco, CA, USA). PJU was a member of the scientific advisory boards of Monogram Biosciences, Inc. (South San Francisco, CA, USA) and XDx, Inc. (Brisbane, CA, USA) and is a cofounder of and consultant to Bayhill Therapeutics (San Mateo, CA, USA). DLT is currently an employee of Genentech, Inc. The other authors declare that they have no competing interests.

## Authors' contributions

DLT conceived of the study idea, contributed to the experimental design, performed experiments, participated in the writing of the manuscript and data interpretation, and helped to perform array studies and conduct statistical analysis. KLG contributed to the experimental design and assisted with animal studies. LYL monitored survival and proteinuria in the mice and assisted with animal studies. IB helped to perform array studies and conduct statistical analysis. PJH contributed to the experimental design and supplied the IFNAR2^-/- ^mice. PJU assisted with conception of the study idea and participated in its design, data analysis, and the writing of the manuscript. All authors read and approved the final manuscript.

## Supplementary Material

Additional data file 1 A table providing a description of the autoantigens used on the protein microarrays, which includes the source, origin, tag information, and known disease associations for each antigen. Click here for file

Additional data file 2 A table containing raw median pixel intensity minus background values for all array antigens and samples used in this study. Click here for file
